# Acute mesenteric ischemia and duodenal ulcer perforation: a unique double pathology

**DOI:** 10.1186/1471-2482-12-21

**Published:** 2012-10-30

**Authors:** Lois Haruna, Ahmed Aber, Farhan Rashid, Marco Barreca

**Affiliations:** 1 Department of General Surgery, Luton and Dunstable Hospital, Lewsey Road, Luton, Bedfordshire, LU4 ODZ, UK

**Keywords:** Acute mesenteric ischaemia, Duodenal ulcer perforation, Mesenteric venous thrombosis, Ischemic bowel infarction, Bowel necrosis

## Abstract

**Background:**

Acute mesenteric ischaemia and duodenal perforation are surgical emergencies with serious consequences. Patients presenting with acute mesenteric ischaemia alone face a high mortality rate as high as 60% whereas those presenting with peptic ulcer perforation the mortality rates range from 6-14%. There are very few reported cases of patients presenting with this dual pathology.

**Case presentation:**

We report a unique case of a 53 year old Italian lady who presented with acute mesenteric ischaemia and duodenal perforation. This is the first report of massive bowel ischaemia and duodenal perforation with no apparent underlying common pathophysiology leading to this presentation.

**Conclusion:**

Early management in the intensive care unit and appropriate surgical intervention maximised the patient’s chances of survival despite the poor prognosis associated with her dual pathology. The rare pathology of the patient described can be explained by two possible hypotheses: peptic ulcer disease causing duodenal ulceration, which precipitated ischaemic infarction of the small bowel. The second hypothesis is the patient developed a stress related ulcer following ischaemic bowel infarction secondary to arterial thrombosis.

## Background

Acute mesenteric ischemia (AMI) comprises a group of pathophysiologic processes that have a common end point—bowel necrosis. The survival rate has not improved substantially during the past 70 years, and the major reason is the continued difficulty in recognizing the condition before bowel infarction occurs [1,2].

Clinical presentation is nonspecific in most cases and can be characterized by an initial discrepancy between severe abdominal pain and minimal clinical findings. In general, patients with AMI have an acute onset of symptoms and a rapid deterioration in their clinical condition. Complications such as ileus, peritonitis, pancreatitis, and gastrointestinal bleeding may also mask the initial signs and symptoms of AMI [2].

Acute mesenteric ischemia can be categorized into 4 specific types based on its cause. The most frequent cause is arterial emboli. They are responsible for approximately 40% to 50% of cases [1,3]. Most mesenteric emboli originate from a cardiac source. The second most common cause is acute mesenteric thrombosis accounts for 25% to 30% of all ischemic events [4,5]. Most of the reported cases of mesenteric ischemia due to arterial thrombosis occur with a background of severe atherosclerotic disease, the most common site near the origin of the Superior Mesenteric Artery [6]. Commonly, patients with this condition can tolerate major visceral artery obstruction because the slow progressive nature of atherosclerosis allows the development of important collaterals. The third major cause is non-occlusive mesenteric ischemia. The pathogenesis of is poorly understood but often involves a low cardiac output state associated with diffuse mesenteric vasoconstriction. Splanchnic vasoconstriction in response to hypovolemia, decreased cardiac output, hypotension, or vasopressors best explain the difference between this entity and other forms of AMI [7,8]. Mesenteric venous thrombosis is the least common cause of mesenteric ischemia, representing up to 10% of all patients with mesenteric ischemia and 18% of those with AMI. Most cases are thought to be secondary to other intra-abdominal pathologic conditions (such as malignancy, intra-abdominal sepsis, or pancreatitis) or were classified as idiopathic [9].

Mesenteric venous thrombosis is usually segmental, with oedema and hemorrhage of the bowel wall and focal sloughing of the mucosa. Thrombi usually originate in the venous arcades and propagate to involve the arcuate channels. Hemorrhagic infarctions occur when the intramural vessels are occluded [2]. Involvement of the inferior mesenteric vein and large bowel is uncommon. The transition from normal to ischemic intestine is more gradual with venous embolism than with arterial embolism or thrombosis.

## Case presentation

A 53-year-old lady presented following a collapse at home. In the ambulance she became unresponsive with a temperature 33.4oC, heart rate of 95 beats per minute and un-recordable blood pressure. On arrival active resuscitation commenced and a brief history of three days of severe, intermittent abdominal pain, absolute constipation, and reduced urine output was noted. Prior to this recent deterioration the patient had been well, with no weight loss or change in appetite. She had a past medical history of hypertension, osteoporosis, a previous appendicectomy; and a 20-pack year smoking history. She denied any dyspeptic symptoms prior to her admission, and had never taken any antacids. Her medications on admission were regular beta blocker, regular long term bisphosphonates and paracetamol on as required basis. On inspection the patient appeared of a normal body habitus, with a BMI of 22 and examination revealed a peritonitic abdomen.

Blood tests from admission revealed grossly elevated inflammatory markers (CRP>500mg/L), neutrophilia and acute kidney failure. Her blood gas showed metabolic acidosis with a high lactate (12.7mmol/L), and low base excess (−21.8 mmol/L) and bicarbonate (6.9mmol/L).

**Figure 1:**
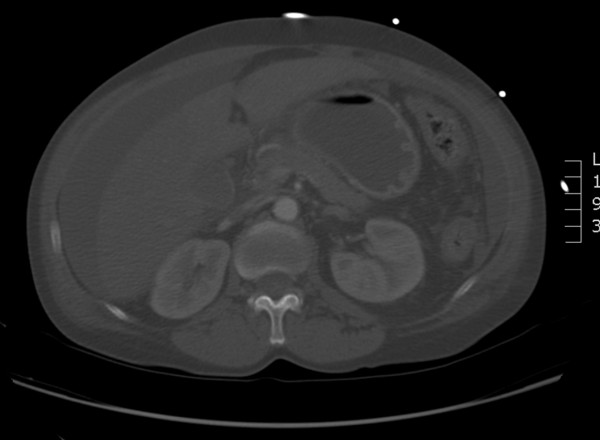
CT scan of the abdomen showing free peritoneal fluid.

Due to her severe acute renal impairment no contrast was initially used for imaging. CT scan of her abdomen performed on admission revealed extensive free peritoneal fluid and also fluid adjacent to the right kidney in the retro peritoneum (Figure 1). There was no evidence of any free air, obstruction, perforation or abdominal aortic aneurysm, with no evidence for small bowel ischaemia. Furthermore an ascitic tap was performed which revealed a clear straw coloured fluid with a very high lactate dehydrogenase, and normal protein. It did not yield growth of any organisms.

Despite optimal management in the intensive care unit the patient’s inflammatory markers, creatinine and alanine aminotranserase all continued to rise, and urine output remained minimal. In light of her clinical deterioration, 12 hours after admission the patient underwent an urgent diagnostic laparoscopy.

Intraoperatively approximately 3 litres of free intraperitoneal purulent bile stained fluid filled the abdomen, and multiple fibrinous exudates surrounded the entire small bowel. A 162 cm section of distal small bowel appeared ischemic, and an anterior duodenal (D1) perforation was identified. The procedure was converted to a laparotomy in view of the findings. The D1 perforation was repaired using an omental patch, and the ischemic bowel wrapped in warm saline-soaked packs, with minimal benefit. The distal 162cm of dead small bowel was resected, and because of her critical condition the neodistal small bowel had a few viable slightly dusky patches left behind for a relook the following day. A washout was performed and a laparostomy using a saline bag was applied.

One day post-operatively the patient was dialysed and kept intubated due to unresolving metabolic acidosis. During the second laparotomy another 50cm of small bowel appeared ischaemia and was resected. 2 meters of healthy small bowel was left in situ. An ileocaecal anastomosis was made, a washout performed, and the abdominal wound closed.

**Figure 2:**
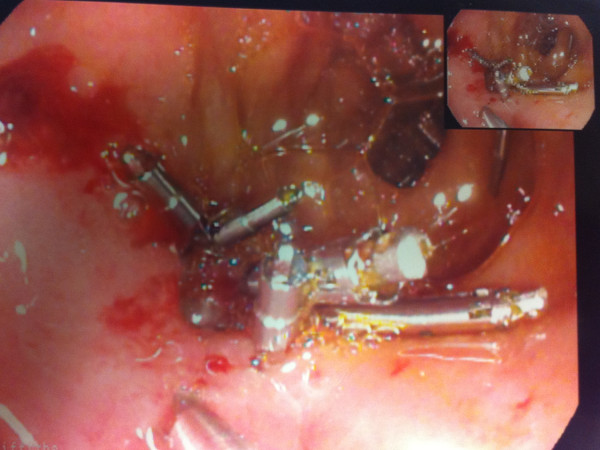
Endoscopic clipping of anterior duodenal perforation.

Two weeks after her initial laparotomy the patient had an endoscopy for a nasojejunal tube insertion. The omental patch repair was performed during laparotomy for duodenal perforated ulcer. However, once endoscopy was performed few days after the first laparotomy for naso-jejunal feeding tube insertion, a small hole was identified near the D1 repair site and was clipped (Figure 2). A repeat endoscopy a week later showed closure of the duodenal defect and a 1cm healing ulcer. The patient made a slowly recovery and she was discharged few weeks later.

Histology showed haemorrhagic infarction in sections of small bowel with ischaemic changes throughout the submucosa and muscle coat, with normal appearing Mesenteric vessels.

## Discussion and conclusion

The unusual presentation of this patient raised an important question regarding her dual pathology: which occurred first? Did she have a perforated duodenal ulcer, causing sepsis and hypotension leading to small bowel ischemia, or did she suffer from ischaemic bowel, and subsequently developed a stress-related perforated ulcer?

After performing a through literature search, we did identify a single case report that described a patient presenting with acute abdomen and the subsequent intervention revealed exactly the same double pathology of Small bowel ischaemia and duodenal ulcer. However the histopathology of the bowel in that particular case did show evidence of polyarteritis nodosa explaining the cause of the acute bowel ischaemia [10].

In order to explain this double pathology it is vital to note that in peptic ulcer disease the two major precipitating factors are Helicobacter pylori infection and non-steroidal anti-inflammatory drugs (NSAIDs). Ulcer incidence increases with age and therapy with drugs such as corticosteroids, anticoagulants and bisphoshonates. Complications (bleeding, perforation, obstruction) can occur in patients with peptic ulcers of any aetiology. Perforation occurs in about 5% to 10% of patients with active ulcer disease [11].

With this background we are proposing two explanations for this pathology. The first is that the patient had been on long term bisphosphonates and this increased her risk for peptic ulcer disease. If we assume that the perforation of the duodenal ulcer occurred first, it was likely that it led to mesenteric venous thrombosis causing ischaemic infarction of the small bowel. The histology results favour this theory as the patient had segmental involvement of the small bowel with the sparing of the large bowel and this commonly present in acute ischaemia of the bowel that is associated with mesenteric venous thrombosis.

The second hypothesis is that the patient developed a stress related duodenal ulcer post ischaemic bowel infarction and eventually this ulcer perforated. The cause of the ischaemia is likely due to arterial thrombosis with a background of severe atherosclerotic disease caused by the patient‘s long history of hypertension and smoking. Patients with this type of bowel ischaemia present later as they can tolerate major visceral artery obstruction because the slow progressive nature of atherosclerosis allows the development of important collaterals. The patient had 3 days history of feeling unwell and constipation with minimal urine output before she collapsed.

## Consent

Written informed consent was obtained from the patient for publication of this Case report and any accompanying images. A copy of the written consent is available for review by the Series Editor of this journal.

## Abbreviations

AMI: Acute mesenteric ischaemia; NSAIDS: Non-steroidal anti-inflammatory drugs.

## Competing interests

The authors declare that they have no competing interests. No financial support has been received.

## Authors’ contribution

LH drafted the manuscript, and conducted a literature search. AA, FR and MB conducted a literature search and contributed to drafting the manuscript. FR monitored the drafting and assisted with the research. MB performed the operation and reviewed the manuscript and gave final approval for publication. All authors read and approved the final manuscript.

## Pre-publication history

The pre-publication history for this paper can be accessed here:

http://www.biomedcentral.com/1471-2482/12/21/prepub
